# Fallville: A Perspective on an Interactive Pedagogical Tool to Enhance Understanding and Implementation of Fall-Compliant Flooring

**DOI:** 10.3390/bioengineering13010080

**Published:** 2026-01-12

**Authors:** Shashank Ghai, Ishan Ghai

**Affiliations:** 1 Department of Political, Historical, Religious and Cultural Studies, Karlstad University, 651 88 Karlstad, Sweden; 2 Centre for Societal Risk Research, Karlstad University, 651 88 Karlstad, Sweden; 3 Independent Researcher, New Delhi 110085, India; ishanghai@live.com

**Keywords:** falls, fall-related injuries, energy dissipation, compliant flooring, gamification

## Abstract

Fall-compliant flooring represents a passive fall preventative approach that has emerged as an effective intervention for reducing fall-related injuries, yet its adoption remains limited due to insufficient understanding among end-users and key stakeholders. To address this knowledge gap, this perspective article provides a proof-of-concept for an interactive pedagogical tool designed to use gamification principles to improve understanding of the mechanical behavior of fall-compliant flooring. This two-part perspective article first establishes the scientific foundation through controlled ball drop experiments comparing energy dissipation between fall-compliant and standard flooring. Through video-based tracking analysis, the experiments quantified kinetic energy and force dissipation across spatial and temporal dimensions. Results revealed that fall-compliant flooring exhibits significantly superior spatiotemporal energy dissipation capabilities compared to standard flooring across both force and kinetic energy metrics. Building on these findings, the second part proposes a conceptual framework for a pedagogical tool that translates these experimental insights into an interactive learning experience that could, in future implementations, allow users to conduct hands-on ball drop activities supported by real-time scientific explanations. This approach transforms complex biomechanical concepts into accessible, engaging learning experiences. By combining experiential learning with gamified elements, this tool, termed “Fallville”, has the potential to increase fall-injury prevention awareness, deepen understanding of fall-compliant flooring mechanisms, and ultimately accelerate adoption of this proven safety intervention in healthcare and residential settings.

## 1. Introduction

Fall-related injuries represent a significant global public health challenge, disproportionately affecting older adults and other vulnerable populations [1,2]. The magnitude of this problem is staggering, with recent statistics indicating that falls account for almost 37 million severe falls yearly that require medical attention, ultimately resulting in 38 million disability-adjusted life years globally every year [3]. The economic burden of falls is equally overwhelming, with treatment costs exceeding $50 billion in the United States and approximately €25 billion in Europe alone [4,5]. This crisis is poised to intensify dramatically as the global population aged 60 and over is projected to reach 2.5 billion by 2050, making the incidence of falls expected to rise exponentially and establishing fall prevention as a critical strategic priority for healthcare systems worldwide [6,7].

### 1.1. Passive Interventions

Despite the development of numerous preventative interventions designed to manage fall injuries, the inevitability of falls due to age-related functional decline and the consistently poor adherence to active participation underscores a pressing need for passive fall-prevention interventions [8,9]. Passive injury prevention measures typically modify the environment and possess greater potential for benefiting the general population since they are not dependent upon engagement or behavioral change, thereby providing automatic protection [10,11,12]. This approach addresses a fundamental challenge in fall prevention: the difficulty individuals, particularly fall-prone older adults, experience in maintaining consistent behavioral changes over time.

Drawing from socioemotional selectivity theory, which suggests that older adults perceive their remaining time as limited, Carstensen and Reynolds [13] propose that this population may prioritize activities offering immediate emotional satisfaction over those perceived as burdensome or time-consuming [14]. Several studies have reaffirmed this theory, reporting difficulties in integrating older adults with active fall prevention strategies into their daily routines primarily because they anticipate short-term social–emotional rewards rather than long-term health benefits [15,16]. This evidence highlights deeper cognitive-motivational barriers among older adults for long-term engagement, revealing a fundamental mismatch between intervention design and the psychosocial realities of aging populations [17,18]. Consequently, there is an imperative for developing and implementing passive interventions, such as fall-compliant flooring systems, that function independently of user compliance, decision-making capacity, or behavioral change requirements.

### 1.2. Fall-Compliant Flooring

Fall-compliant flooring represents a promising passive intervention that reduces fall-related injuries by reducing impact forces through two principal mechanisms: reducing ground stiffness and dissipating energy upon impact, thereby lowering reactionary forces on the falling body and reducing injury severity [19]. Extensive biomechanical research over the past decade has demonstrated that fall-compliant flooring produces substantially lower impact forces than conventional surfaces, resulting in reduced fall-injury risk [19,20,21,22,23]. Importantly, several studies in long-term care facilities have shown measurable reductions in fall-related injuries [24,25,26], which provides real-world evidence of the effectiveness of this passive preventative approach.

However, widespread adoption of fall-compliant flooring remains limited, likely because it is a relatively new intervention and due to insufficient understanding of its functional mechanisms among key stakeholders and end users [27,28]. Previous studies attempting to address this knowledge gap reveal methodological limitations in information delivery approaches. For instance, Lachance and Mackey used passive podium presentations at a one-day symposium; although the authors did include interactive workshops with a workshop facilitator to ensure contribution after the podium presentations, this approach may have restricted comprehensive understanding [27]. Similarly, Kalra and colleagues surveyed stakeholders who experienced the flooring firsthand [29]. While both studies are innovative in their approaches to stakeholder engagement, they lack structured pedagogical approaches capable of deepening conceptual understanding regarding the underlying mechanics. This gap points to the need for knowledge-translation strategies that not only inform stakeholders but also support conceptual engagement with the underlying mechanics of fall-compliant flooring. Addressing this knowledge gap requires instructional approaches grounded in established learning theories.

### 1.3. Pedagogical Innovation for Knowledge Translation

Active pedagogical strategies, particularly those incorporating active learning, experiential learning, and gamification, may provide an effective mechanism for deepening stakeholder and end-user understanding of fall-compliant flooring. Active learning theories emphasize that learners construct understanding through participation, inquiry, and reflection rather than passive reception of information [30,31,32]. This aligns with constructivist principles [33], which posit that learning occurs when individuals interact with physical phenomena and socially mediate problem-solving tasks. Similarly, Merrill [34]’s First Principles of Instruction highlight the importance of engaging learners in real-world problems, activating prior knowledge as a foundation for new knowledge, demonstration, application, and integration, principles ideally suited for teaching complex biomechanical concepts such as energy dissipation.

Gamification can further strengthen engagement and motivation in such a pedagogical approach, particularly for stakeholders without technical backgrounds. Motivational design frameworks such as those by Deterding, Dixon [35], Kapp [36], and Werbach and Hunter [37] emphasize mechanisms such as challenge, feedback loops, narrative framing, and meaningful goal structures. These align functionally with Self-Determination Theory, which identifies development of competence, autonomy, and relatedness as central drivers of intrinsic motivation [38]. Together, these frameworks suggest that gamified, experiential instruction could make the mechanics of fall-compliant flooring both accessible and engaging.

Building on this, a pedagogical tool that enables users to walk on different floors, conduct object-drop experiments, and analyze impact behavior would situate learning within hands-on, inquiry-driven tasks, consistent with Kolb’s experiential learning cycle [39]. By progressing through concrete experience, reflective observation, abstract conceptualization, and active experimentation, learners could potentially gain a more intuitive understanding of energy-dissipation principles. This form of experiential and gamified engagement could also strengthen knowledge translation, as outlined in the Knowledge-to-Action framework [40], by transforming complex biomechanical concepts into practical, accessible insights that can meaningfully inform stakeholder decision-making and facilitate end-user adoption.

This article represents a first step in introducing such a pedagogical tool designed to enhance stakeholder and end-user engagement with fall-compliant flooring. The broader intent is to provide a platform that bridges scientific evidence with user understanding, engaging relevant professional stakeholders, end users, and the general population in actively learning, through first-principles physics, how energy dissipation works in fall-compliant flooring and its relationship to fall-related injury prevention, thereby fostering broader adoption [41].

### 1.4. Objectives

In this two-part perspective article, the goal is to present a comprehensive approach to improving understanding and adoption of fall-compliant flooring systems.

Part-1 presents experimental findings comparing the energy dissipation properties of a commercially available fall-compliant sports floor with standard flooring using ball-drop experiments in a controlled and calibrated setting. These experiments will provide empirical evidence that will essentially be included in Part-2 to make a pedagogical tool that can educate a diverse audience concerning fall energy dissipation.

Part-2 proposes a conceptual framework for a prospective gamified pedagogical tool that could incorporate these experiments to enhance stakeholder and end-user understanding of fall-compliant flooring mechanics and the functioning of fall-compliant flooring systems in injury reduction.

## 2. Part-1: Experimental Foundation

### 2.1. Methods

#### 2.1.1. Equipment

In a calibrated setting, ball drop experiments were conducted using three balls with different weights to evaluate the energy dissipation properties of a fall-compliant flooring of 40 mm thickness (Pusselmatt, Nordic Fighter, Finnerödja, Sweden). As the study involved only controlled drops of inanimate objects and did not include any human participation, ethical approval was therefore not required.

The three balls used in the experiments varied in mass and size: a tennis ball (mass: 56 g, radius: 3.2 cm), a table tennis ball (mass: 2.7 g, radius: 1.9 cm), and a rubber ball (mass: 13.7 g, radius: 1.7 cm). The material composition of the balls was not specified on their packaging. However, conventionally, table tennis balls are made from acrylonitrile butadiene styrene [42], tennis balls have a pressurized rubber core with a wool and nylon felt outer layer [43], and rubber balls are typically made primarily from cis-poly (1, 4-isoprene) and other forms of synthetic rubbers [44]. The experimental setup has been illustrated in Supplementary Figure S1. Videos were recorded using a 12-megapixel camera at 60 frames per second on an iPhone 12 (Apple Inc., Cupertino, CA, USA), which was placed on an iPhone stand at a distance of 43 cm. The data was then subjected to post-processing using the open-source video analysis software Tracker (Version 6, Physlets).

#### 2.1.2. Procedure and Data Analysis

To facilitate calibration and ensure consistent dropping height during video analysis, a marker was placed on a wall at a distance of 0.8 m from the floor in a well-lit environment. This ensured accurate measurement of the falling object’s trajectory. A smartphone was positioned on a stand, and different balls were dropped on both fall-compliant and standard flooring. All videos were recorded for 10 s. Thereafter, the videos were imported to a computer and analyzed using the Tracker software. The requisite pre-processing was conducted by aligning the calibration stick with the marked distance of 0.8 m from the ground. Subsequently, a point mass object was marked on the surface of the ball, and the recommended auto-tracking function was selected for the purpose of tracking the trajectory of the dropped ball. The selected template was set with an evolution rate of 20% and a tether rate of 5%. In instances where the auto-tracking function was unable to track the ball with sufficient precision, manual marking was performed to ensure accuracy. The video recordings of the complete tracking procedure for the ball drop experiments are provided in the Supplementary File S2.

The dissipation of energy during the ball drop experiment was studied by evaluating the changes in the force (SI unit: newton). This was calculated by multiplying the mass (kilogram) of the balls by the observed acceleration (meter/second^2^).

$$1\;Newton=1\;kilogram\times meter\times {second}^{-2}$$

Force=mass×acceleration

In addition, kinetic energy, whose SI unit is expressed in joules, was also evaluated. Both the acceleration and kinetic energy parameters were extracted from the automatic computational option available in the Tracker software.

$$1\;Joule=1\;kilogram\times {meter}^{2}\times {second}^{-2}$$

$$Kinetic\;energy=\frac{1}{2}mass\times {velocity}^{2}$$

The temporal dissipation of overall force and kinetic energy was illustrated by plotting the measurements over the 10 s period. Similarly, the spatial dissipation of force and kinetic energy was demonstrated by plotting these measurements from the initial drop height (i.e., 0.8 m) and subsequent rebounds until the ball came to rest. Descriptive statistics were computed for all balls within each ball drop condition. Moreover, a paired *t*-test was conducted to compare differences in kinetic energy and force for each ball drop condition, separately on both standard and fall-compliant flooring. For the assessment of test–retest reliability of the video tracking system, data from five separate ball drop experiments were analyzed. In each trial, the same ball was used for multiple drops, ensuring consistency across trials. The recording duration for each drop was held constant, which corresponded to the time period from the moment the ball was released until it came to rest. To evaluate the reliability of the tracking, the Intraclass Correlation Coefficient (ICC) was calculated using a two-way mixed-effects model. All analyses were performed using IBM SPSS Version 29.0 (Armonk, NY, USA).

### 2.2. Part-1 Results

#### 2.2.1. Test–Retest Reliability

The analysis of test–retest reliability was performed by dropping a table tennis ball onto a fall-compliant floor on five different occasions. The datasets are provided in the Supplementary Table S1. The analysis revealed a single measure ICC of 0.68 (95% CI: 0.60 to 0.74), indicating moderate to good reliability for individual trials. The average measure ICC was 0.91 (95% CI: 0.88 to 0.93), indicating excellent reliability when multiple trials were averaged. The F-test showed statistically significant reliability, F_108, 432_ = 11.61, *p* < 0.001. These results suggest that the tracking system provides consistent measurements.

#### 2.2.2. Spatiotemporal Dissipation

**Figure 1:**
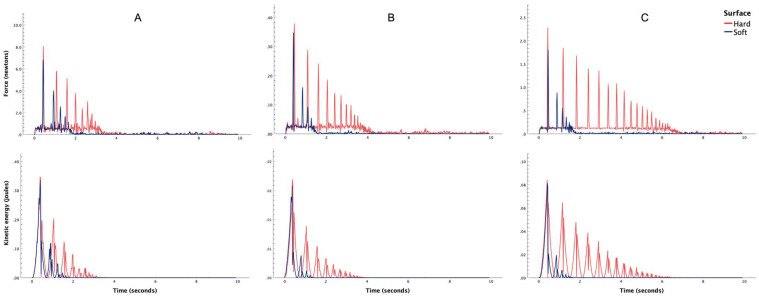
Temporal changes in force (newtons) kinetic energy (joules) during the ball drop experiment on fall-compliant (soft) and standard (hard) flooring with (**A**) tennis ball, (**B**) table tennis ball, (**C**) rubber ball.

**Figure 2:**
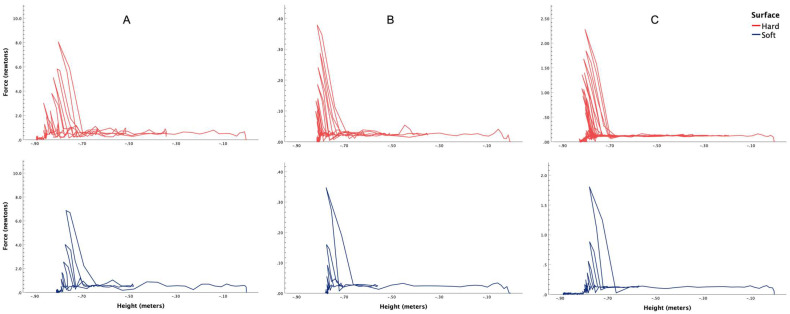
Spatial changes in force (newtons) during the ball drop experiment on fall-compliant (soft) and standard (hard) flooring with (**A**) tennis ball, (**B**) table tennis ball, (**C**) rubber ball.

**Figure 3:**
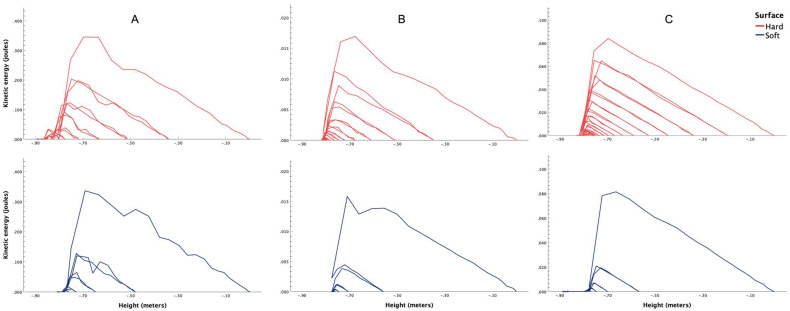
Spatial changes in kinetic energy (joules) during the ball drop experiment on fall-compliant (soft) and standard (hard) flooring with (**A**) tennis ball, (**B**) table tennis ball, (**C**) rubber ball.

Figure 1 shows the temporal dissipation of total force and kinetic energy for all three balls, i.e., tennis (Figure 1A), table tennis (Figure 1B) and rubber ball (Figure 1C). The spatial dissipation of force is shown in Figure 2 for tennis (Figure 2A), table tennis (Figure 2B) and rubber ball (Figure 2C). Similarly, the spatial dissipation of kinetic energy has been illustrated in Figure 3 for tennis (Figure 3A), table tennis (Figure 3B) and rubber ball (Figure 3C).

##### Tennis Ball

On the standard flooring, the tennis ball experienced a mean (± standard deviation) kinetic energy of 4.2 × 10^−2^ ± 6.3 × 10^−3^ joules, compared to 2.4 × 10^−2^ ± 6.0 × 10^−3^ joules on the fall-compliant floor. A paired *t*-test revealed significantly higher kinetic energy on the standard flooring as compared to fall-compliant flooring, t_211_ = 6.5, *p* < 0.001.

The ball also experienced greater force on the standard floor, 8.0 × 10^−1^ ± 1.0 newtons, compared to the compliant floor, 4.7 × 10^−1^ ± 8.8 × 10^−1^ newtons, with this difference also reaching significance, t_211_ = 4.7, *p* < 0.001.

##### Table Tennis Ball

On the standard flooring, the table tennis ball experienced a mean kinetic energy of 1.7 × 10^−3^ ± 2.9 × 10^−3^ joules, compared to 8.0 × 10^−4^ ± 2.4 × 10^−3^ joules on the fall-compliant floor. A paired *t*-test revealed significantly higher kinetic energy on the standard flooring, t_253_ = 6.8, *p* < 0.001.

The ball also experienced greater force on the standard floor, 3.5 × 10^−2^ ± 4.8 × 10^−2^ newtons, compared to the compliant floor, 1.4 × 10^−2^ ± 3.4 × 10^−2^ newtons, with this difference also reaching significance, t_253_ = 6.1, *p* < 0.001.

##### Rubber Ball

On the standard flooring, the rubber ball experienced a mean kinetic energy of 9.6 × 10^−3^ ± 1.4 × 10^−2^ joules, compared to 2.5 × 10^−3^ ± 9.8 × 10^−3^ joules on the fall-compliant floor. A paired *t*-test revealed significantly higher kinetic energy on the standard flooring, t_421_ = 11.8, *p* < 0.001.

The ball also experienced greater force on the standard floor, 1.8 × 10^−1^ ± 2.6 × 10^−1^ newtons, compared to the compliant floor, 5.3 × 10^−2^ ± 1.4 × 10^−1^ newtons, with this difference also reaching significance, t_421_ = 10.2, *p* < 0.001.

### 2.3. Part-1 Discussion

This controlled study utilized a ball drop experiment with video tracking analysis to compare the spatiotemporal dissipation of energy and force between fall-compliant and standard flooring. The results consistently demonstrated that fall-compliant flooring outperformed standard flooring in dissipating energy, irrespective of the ball type used (tennis, table tennis, or rubber). Specifically, the fall-compliant flooring reduced overall force (Figure 2) and kinetic energy (Figure 3), while accelerating the dissipation of these parameters over time (Figure 1). From a biomechanical perspective, these results provide crucial insights into the injury mitigation mechanisms of fall-compliant flooring. During falls, excessive kinetic energy and impact forces can exceed anatomical and physiological tolerance thresholds, leading to fractures or soft tissue damage [45].

To understand these protective mechanisms, it is essential to consider the fundamental biomechanics underlying fall injury prevention, i.e., the transfer of kinetic energy from the falling object to the surface upon impact and the resulting reactionary forces. Based on our results, we propose that fall-compliant flooring’s ability to deform under impact resulted in a prolonged deceleration phase, thereby altering the force-time relationship during collision. According to the impulse-momentum theorem, extending the contact duration necessarily reduces impact forces for a given momentum change. This temporal redistribution of force represents a primary protective mechanism, as biological tissues exhibit strain-rate dependent failure properties, meaning, higher loading rates produce higher stresses and greater injury risk.

Our findings support this biomechanical interpretation. The ball drop experiments revealed significantly higher mean kinetic energies and forces on standard flooring as compared to fall-compliant flooring. For instance, in terms of mean kinetic energy, balls experienced 75 to 284% higher values on standard flooring compared to fall-compliant flooring, with a corresponding 70 to 240% increase in mean force. These reductions in loading rate have biomechanical relevance for bone and soft tissue, potentially decreasing the likelihood of contusions, hematomas, and other impact-related injuries. Since tissue injury occurs when local stresses exceed anatomical failure thresholds, lowering impact forces and loading rates may reduce the probability that real-world falls surpass these critical limits.

The material properties of fall-compliant flooring, including elastic modulus, damping coefficient, and thickness, work synergistically to achieve this protective response. When impact occurs on a deformable surface, the material’s compliance allows for gradual energy absorption, effectively distributing the impact load over a longer time period and increasing the contact area. The initial elastic compression provides immediate force attenuation, while the flooring’s damping characteristics dissipate stored energy rather than returning it to the impacting body. This viscoelastic behavior explains why fall-compliant flooring not only reduces forces but also limits the magnitude of subsequent rebounds, as evidenced by the temporal dissipation patterns in Figure 1. In contrast, rigid surfaces undergo minimal deformation, rapidly transmitting energy back to the falling object, resulting in near-complete energy return manifested as multiple high-energy rebounds. The biomechanical consequence is that rigid surfaces concentrate impact forces both spatially and temporally, creating stress magnitudes that exceed tissue failure limits, ultimately causing injury. The extended rebound durations and number of successive bounces demonstrate that rigid surfaces maintain hazardous energy levels for substantially longer periods, whereas fall-compliant flooring effectively attenuates this residual energy.

Furthermore, the present study’s approach to measuring spatiotemporal energy dissipation represents an advancement over previous methodologies, particularly those by Crane, Goodworth [46]. While these authors examined energy absorption by comparing first rebound heights to initial drop heights, their approach captured only a single energy transfer event. In contrast, our methodology quantifies the full sequence of post-impact rebounds, revealing that objects experience multiple rebounds on standard flooring, with each subsequent impact involving substantial reactionary forces. This analysis is particularly significant because it quantifies the repetitive reactionary forces that contribute substantially to fall-related injuries. By characterizing energy dissipation over extended time periods rather than focusing on isolated impact events, this methodology provides a more comprehensive understanding of spatiotemporal dissipation of kinetic energy and force. Additionally, the spatiotemporal analysis framework developed in this study could prospectively serve as an accessible, low-cost, standardized testing tool for evaluating different fall-compliant flooring systems. Furthermore, the consistency of protective effects across balls with different masses and material properties indicates that the protective mechanism operates effectively across diverse impact scenarios.

#### Part-1 Limitations

Despite these contributions, several methodological considerations regarding the ball drop experiments warrant acknowledgment. The calibrated experimental setup, while enabling rigorous measurement, does not fully replicate real-world fall scenarios where falls occur under diverse and unpredictable conditions [26,47]. The use of balls to illustrate energy dissipation, though effective for demonstrating principles of energy transfer in a simplified manner, does not capture the full biomechanical complexity of human falls [48,49]. The material composition, mass distribution, and mechanical properties of the human body differ substantially from those of the balls used, particularly when considering older adults with compromised musculoskeletal systems due to osteoporosis. This represents an inherent simplification of the complex biomechanics involved in real-world fall scenarios. Additionally, it is important to consider potential negative effects of fall-compliant flooring. Clinical evidence suggests a potential trade-off: while fall-compliant flooring may reduce injury severity, it may also increase fall risk due to balance challenges introduced by the softer material [50,51,52], underscoring the need for comprehensive testing methodologies [19,53].

Technical limitations could have also influenced data acquisition precision in our experiment. The absence of force plate measurements, due to infrastructure limitations at our facility, prevented direct and validated comparative measurements of actual forces transmitted during impact [54]. Additionally, the study relied on video analysis using a smartphone camera at 60 fps. While this approach is cost-effective and accessible, it lacks the precision of sophisticated motion capture systems (≥500 frames per second). The 60 fps sampling frequency could have limited the temporal resolution of kinematic data [55], particularly during the brief impact phase. This constraint may have resulted in underestimation of the true peak values and reduced precision in force calculations, as the exact moment of maximum deceleration may fall between sampled frames.

Despite these limitations, it is important to highlight that the excellent test–retest reliability (ICC = 0.91) demonstrates the consistency of our measurement approach. Importantly, the findings provide valuable proof-of-concept data demonstrating how low-cost modalities using simple ball drop experiments can foster experiential understanding of energy dissipation principles. Thus, this approach primarily supports the pedagogical tool’s educational objectives rather than serving as a precise biomechanical evaluation of human falls.

Having established the empirical foundation through controlled experiments in Part-1, the following section presents a conceptual framework of a pedagogical tool for translating these findings into an interactive learning experience.

## 3. Part-2 Discussion: Elaborating a Perspective on the Development of the Pedagogical Tool

In the existing state of literature, educational and gamification approaches have widely demonstrated effectiveness in enhancing practical understanding of complex scientific concepts [56,57,58]. These methodologies have been shown to encourage critical thinking and discussion, ultimately improving preparedness and resilience when addressing real-world challenges [56,59].

This article, while primarily focused on comparing spatiotemporal energy dissipation between fall-compliant and standard flooring, was developed as part of a prospective pedagogical tool called “Fallville.” Drawing on insights from the ball drop experiments, the goal of this pedagogical tool is to enhance understanding of fall-compliant flooring performance among a broad audience, including stakeholders, end users, and perhaps even the general population. Fallville’s approach incorporates several pedagogical considerations. For instance, the open-source video tracking system ensures technological accessibility, while real-time analysis capabilities allow immediate interpretation of ball drop experiments on different fall-compliant and standard floors during demonstrations. Moreover, the use of balls with varying material compositions is intended to demonstrate fall-compliant flooring’s energy dissipation effectiveness across different scenarios, providing participants with tangible evidence of the technology’s protective capabilities.

This dual-purpose approach aims to integrate rigorous scientific methodology with educational accessibility, creating a foundation for both research advancement and knowledge dissemination. The Fallville concept is intended to transform complex biomechanical principles involved in the field of fall mechanics into observable, understandable demonstrations that can inform decision-making processes across multiple stakeholder groups. The section below discusses how this tool may engage participants in a step-by-step manner.

### 3.1. Prospective Steps for Playing Fallville

**Figure 4:**
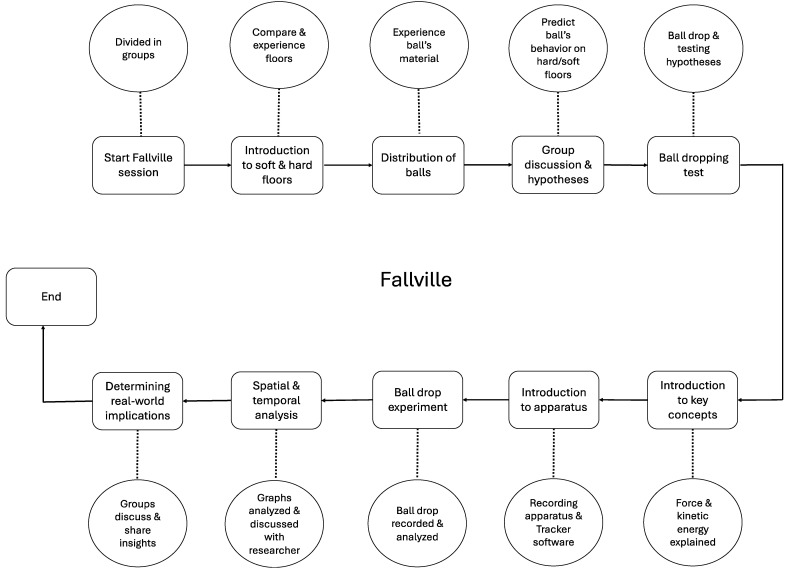
Schematic flow of the proposed Fallville tool.

Fallville has been designed for use by a broad range of individuals, including expert stakeholders, end users, and even the general population, meaning that no prior knowledge of fall mechanics is required. The prospective design of the Fallville tool involves a stepwise approach illustrated in Figure 4.

Each 20 to 30 min session would begin with participants exploring the fall-compliant flooring by comparing its stiffness, grip, and texture to standard hard flooring through hands-on experience. Participants would then be provided with balls made from different materials to allow them to experience variations in material behavior. Before testing, they would be encouraged to discuss and predict how the different balls might behave when dropped on both standard and fall-compliant flooring. After sharing their hypotheses, they would perform the drops and observe the outcomes, testing their predictions. Following this, the researcher would then provide a brief introduction to key concepts such as force and kinetic energy, and their relevance to fall injury prevention. The session would conclude with an open discussion, where participants could ask questions and further explore the presented concepts.

Participants would then be introduced to a calibrated experimental setup, including a smartphone mounted on a stand and connected to a laptop running Tracker software. They would drop the balls from a marked height onto both standard and fall-compliant floors. The events would be recorded, and the videos analyzed using the software’s automatic tracking feature, which would generate spatiotemporal graphs of energy dissipation through pre-built modules. Participants would then be asked to interpret these graphs (Figure 1, Figure 2 and Figure 3) and discuss their thoughts with the researcher. The researcher would also offer a detailed explanation of the observed spatiotemporal changes and address any questions. After these discussions, participants would also be encouraged to reflect on and report the potential real-world implications of their findings.

### 3.2. Other Prospective Uses of Fallville

In addition to fostering understanding and encouraging the adoption of fall-compliant flooring among key stakeholders, Fallville could serve as a valuable research communication tool. Its accessibility, ease of implementation, and cost-effectiveness could make it well suited for engaging participants from diverse backgrounds. One of its core elements, assessing energy dissipation, is grounded in fundamental physics concepts such as kinetic energy and force, which are commonly taught in educational curricula. Therefore, its prospective use in higher education settings to help students from medical backgrounds develop an understanding of fall mechanics from first principles could be of significant value.

Furthermore, Fallville has the potential to raise public awareness about the prevalence and consequences of falls and related injuries, prompting participants to critically examine the mechanics underlying the complex nature of falls. By investigating a multitude of factors influencing falls and formulating hypotheses about the impact of falls on various surfaces, participants could gain deeper insights into the real-world implications of energy dissipation. This hands-on approach would enable participants to interpret fall-related injuries in new and meaningful ways.

Looking ahead, Fallville sessions could also provide opportunities to gather participant feedback and reflections, helping to guide iterative improvements to the tool. Over time, this evolving educational resource could further enhance public engagement with injury prevention research, while supporting broader dissemination of knowledge about the protective potential of fall-compliant flooring.

### 3.3. Part-2 Limitations

Several limitations specific to the Fallville pedagogical tool concept warrant acknowledgment. As this is a perspective article proposing a conceptual framework, it is important to consider that the tool has not been empirically tested in pilot or exploratory settings with actual participants. The anticipated educational impact, stakeholder engagement, and effectiveness in promoting understanding of fall-compliant flooring remain entirely theoretical and speculative. Without implementation studies, it is possible that we are overestimating the tool’s potential to influence knowledge, attitudes, and adoption decisions regarding fall-compliant flooring. The extent to which participants would find the activities engaging, comprehend the scientific concepts presented, or translate this understanding into real-world decision-making remains unknown.

Additionally, practical implementation barriers have not been systematically evaluated. The tool requires specific equipment (fall-compliant flooring samples, standard flooring, various balls, smartphone, stand, laptop with Tracker software) and a calibrated setup, which may limit accessibility for potential users. The technical expertise required to operate the video tracking software and interpret spatiotemporal graphs may also present challenges for facilitators. Future empirical studies with formal participant assessments are essential to validate the tool’s educational effectiveness and practical feasibility.

## 4. Conclusions

This two-part article first examined the energy dissipation properties of fall-compliant flooring in comparison to standard flooring through a series of ball drop experiments. The findings indicate that both kinetic energy and force are consistently and significantly lower when all three balls are dropped onto fall-compliant flooring. In addition, energy dissipation occurs more rapidly on the fall-compliant surface compared to the standard floor.

In its second part, the article introduced an educational tool, Fallville, designed to engage stakeholders and end users in understanding the principles and benefits of fall-compliant flooring. The tool builds on the experimental findings of the ball drop experiments and presents them in a stepwise, pedagogical format to stimulate discussion around the mechanics of fall-related injuries and their prevention. A step-by-step guide is provided to support the delivery of Fallville sessions. By encouraging reflection, critical thinking, and dialogue, Fallville has the potential to serve as an effective communication bridge between scientific research and the wider public, promoting greater awareness of fall-related risks and injury prevention strategies [60,61].

At present, Fallville is being implemented in our laboratory and educational environment, with the goal of raising awareness about the importance of fall-compliant flooring and fostering informed discussions about injury prevention across a range of settings. Looking ahead, this tool could also contribute to a broader public understanding of the biomechanical principles underlying falls and support the wider adoption of injury prevention interventions in healthcare, education, and community environments.

## 5. Future Directions

**Figure 5:**
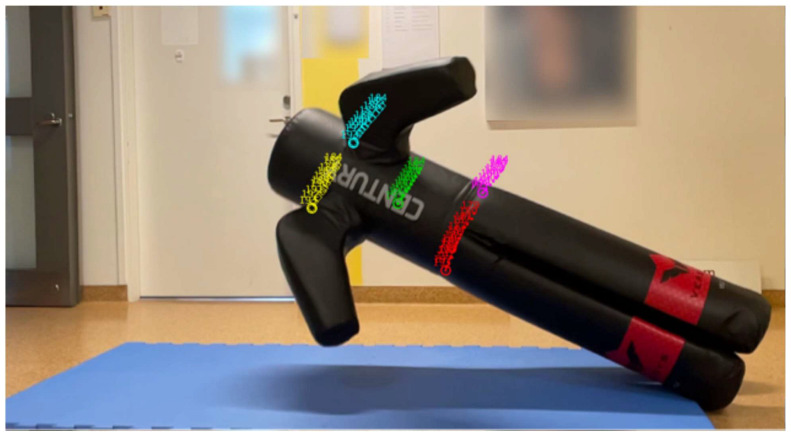
Prospective use of a humanlike dummy in future versions of Fallville for assessing energy dissipation on fall-compliant surfaces. Marker colors: red and pink for hips, yellow and turquoise for shoulders, green for the center of mass of the dummy. Numbers denote individual frames.

Future research should aim to address these limitations by incorporating more comprehensive and realistic fall simulations. One promising approach is the use of advanced biomechanical models that can better reflect the dynamics of real-world falls. The integration of anthropomorphic test devices, such as crash test dummies or humanlike dummies, could support a more accurate assessment of the energy dissipation properties of fall-compliant flooring. These models can simulate a wide range of body movements and fall directions, providing deeper insights into injury mechanisms and the protective benefits of various flooring systems. Currently, the energy dissipation properties of a humanlike dummy (Century Versys VS.2, Germany) are being tested for possible inclusion in future versions of Fallville (Figure 5).

To further improve the accuracy and precision of energy dissipation data, it is also essential to integrate advanced technologies such as high-speed cameras, force plates, and three-dimensional motion analysis systems. These tools would enable a more detailed understanding of the spatial and temporal aspects of energy transfer during falls and would enhance the reliability and validity of the results. In addition, to further enrich the educational and gamification aspects of Fallville, future iterations could include a wider variety of test objects. These could involve balls or other surrogates made from biomaterials that more closely resemble human tissues, such as muscle or bone. This would help participants better understand how energy dissipation differs between various flooring materials and how it relates to specific body parts, thereby improving their ability to predict real-world outcomes. Incorporating a broader range of fall-compliant flooring options, with varying thicknesses, densities, and material compositions such as foam or gel, would also allow for a more comprehensive evaluation of energy dissipation across different surface types.

Finally, it is important to validate the effectiveness of Fallville in promoting participants’ understanding of fall risk and injury prevention. Future studies should assess participants’ knowledge and beliefs both before and after engaging with the tool, using established instruments such as the Generic Workshop Appraisal Scale [62], the User Experience of Safety Questionnaire [29], and even the Fall Prevention Knowledge test [63].

## Data Availability

The raw data from the ball drop experiments will be made available by the authors on request.

## Supplementary Materials

The following supporting information can be downloaded at https://www.mdpi.com/article/10.3390/bioengineering13010080/s1. Supplementary File S1 contains Table S1: Data used for assessing test-retest reliability; Figure S1: Experimental setup for the ball drop experiment; Supplementary File S2 contains Video S1: Tennis ball dropped on hard surface; Video S2: Tennis ball dropped on soft surface; Video S3: Table tennis ball dropped on hard surface; Video S4: Table tennis ball dropped on soft surface; Video S5: Bouncy ball dropped on hard surface; Video S6: Bouncy ball dropped on soft surface.
