# Supplementary material for: Fallville: A Perspective on an Interactive Pedagogical Tool to Enhance Understanding and Implementation of Fall-Compliant Flooring
Source: Bioengineering (Basel). 2026 Jan 12;13(1):80. doi: 10.3390/bioengineering13010080 (PMC12838354; doi:10.3390/bioengineering13010080)
Supplement: Supplementary file 1 [file bioengineering-13-00080-s001.zip › Supplementary file Bioengineering S1.pdf]

Fallville: A Perspective on an Interactive Pedagogical Tool to Enhance Understanding and Implementation of Fall-Compliant Flooring

Supplementary File

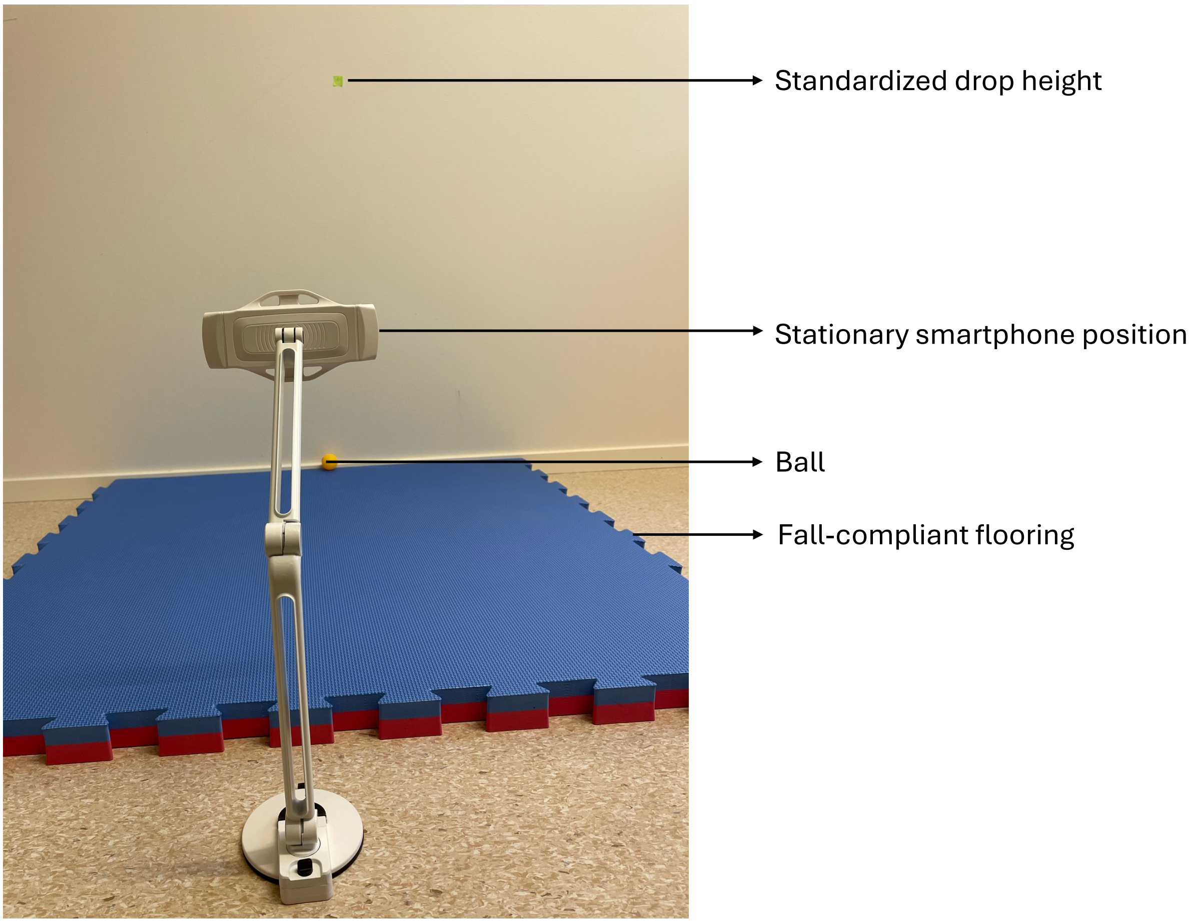

Figure S1. Experimental setup for the ball drop experiment

Table S1. Data used for assessing test-retest reliability

Time	Kinetic energy				
	Drop 1	Drop 2	Drop 3	Drop 4	Drop 5
0.02	0.00	0.00	0.02	0.00	0.00
0.03	0.00	0.00	0.07	0.02	0.00
0.05	0.00	0.00	0.16	0.05	0.00
0.07	0.01	0.03	0.30	0.07	0.00
0.08	0.02	0.09	0.49	0.16	0.00
0.10	0.07	0.19	0.76	0.27	0.00
0.12	0.10	0.40	0.95	0.44	0.00
0.13	0.12	0.60	1.17	0.62	0.00
0.15	0.18	0.76	1.48	0.84	0.07
0.17	0.30	1.06	1.75	1.13	0.14
0.18	0.49	1.40	2.04	1.46	0.16
0.20	0.65	1.74	2.43	1.83	0.27
0.22	0.89	2.18	2.91	2.10	0.42
0.23	1.11	2.63	3.28	2.42	0.59
0.25	1.42	3.06	3.80	2.91	0.82
0.27	1.76	3.57	4.32	3.33	1.15
0.28	2.00	4.03	4.85	3.88	1.41
0.30	2.39	4.65	5.40	4.40	1.69
0.32	2.84	5.26	5.93	4.81	2.06
0.33	3.19	5.73	6.58	5.38	2.53
0.35	3.68	6.31	7.24	5.97	2.99
0.37	4.16	6.89	7.89	6.85	3.51
0.38	4.56	7.45	8.62	7.52	4.01
0.40	5.20	8.06	9.43	8.14	4.32
0.42	5.88	8.94	4.37	9.21	4.98
0.43	6.41	9.92	0.12	9.93	5.70
0.45	7.15	5.50	3.01	5.16	6.24
0.47	7.76	0.05	2.36	0.16	6.90
0.48	8.52	3.02	1.63	3.33	7.60
0.50	9.43	2.30	1.30	2.19	8.20
0.52	4.13	1.68	1.06	2.04	9.13
0.53	0.15	1.26	0.78	1.64	10.10
0.55	2.41	1.00	0.54	1.27	3.39
0.57	1.85	0.74	0.34	0.97	0.45
0.58	1.54	0.49	0.21	0.72	3.38
0.60	1.23	0.31	0.09	0.48	2.44
0.62	0.98	0.20	0.03	0.29	1.58

0.63	0.73	0.10	0.01	0.18	1.56
0.65	0.45	0.03	0.03	0.07	1.24
0.67	0.27	0.01	0.07	0.01	0.91
0.68	0.16	0.01	0.16	0.00	0.66
0.70	0.07	0.05	0.30	0.04	0.45
0.72	0.02	0.12	0.48	0.13	0.26
0.73	0.01	0.24	0.70	0.28	0.13
0.75	0.05	0.42	0.88	0.36	0.11
0.77	0.13	0.68	1.16	0.52	0.05
0.78	0.24	0.93	1.48	0.82	0.01
0.80	0.40	1.20	1.81	1.02	0.07
0.82	0.56	1.55	2.17	1.30	0.15
0.83	0.80	1.93	2.18	1.65	0.25
0.85	1.15	2.11	0.14	1.97	0.37
0.87	1.47	2.56	0.59	2.38	0.55
0.88	1.81	0.87	0.57	2.32	0.85
0.90	2.20	0.14	0.40	0.08	1.34
0.92	2.61	0.76	0.24	0.85	1.83
0.93	1.33	0.60	0.12	0.65	1.96
0.95	0.03	0.42	0.05	0.44	2.22
0.97	0.76	0.28	0.01	0.26	2.68
0.98	0.54	0.13	0.01	0.14	2.25
1.00	0.35	0.05	0.04	0.05	0.02
1.02	0.19	0.04	0.12	0.01	1.08
1.03	0.09	0.07	0.22	0.01	0.85
1.05	0.03	0.16	0.36	0.04	0.44
1.07	0.01	0.28	0.57	0.12	0.29
1.08	0.04	0.43	0.85	0.24	0.19
1.10	0.11	0.62	0.08	0.38	0.06
1.12	0.22	0.81	0.17	0.58	0.02
1.13	0.39	1.13	0.18	0.82	0.02
1.15	0.60	0.39	0.09	0.25	0.07
1.17	0.86	0.08	0.01	0.08	0.03
1.18	1.04	0.22	0.01	0.22	0.05
1.20	0.09	0.11	0.03	0.11	0.26
1.22	0.26	0.04	0.08	0.03	0.39
1.23	0.16	0.01	0.19	0.01	0.59
1.25	0.06	0.03	0.16	0.02	0.88
1.27	0.03	0.08	0.00	0.08	0.41
1.28	0.03	0.17	0.08	0.17	0.06
1.30	0.09	0.29	0.02	0.28	0.33
1.32	0.18	0.17	0.00	0.03	0.19

1.33	0.34	0.03	0.02	0.09	0.05
1.35	0.35	0.11	0.09	0.04	0.01
1.37	0.03	0.04	0.03	0.00	0.03
1.38	0.09	0.03	0.01	0.01	0.07
1.40	0.04	0.08	0.01	0.06	0.13
1.42	0.02	0.18	0.00	0.04	0.26
1.43	0.06	0.08	0.02	0.02	0.28
1.45	0.16	0.05	0.00	0.02	0.04
1.47	0.11	0.05	0.01	0.01	0.13
1.48	0.02	0.06	0.02	0.02	0.07
1.50	0.04	0.16	0.01	0.01	0.04
1.52	0.04	0.12	0.01	0.02	0.05
1.53	0.08	0.05	0.01	0.01	0.11
1.55	0.05	0.06	0.00	0.01	0.08
1.57	0.04	0.09	0.00	0.01	0.05
1.58	0.04	0.17	0.00	0.01	0.08
1.60	0.07	0.15	0.01	0.02	0.05
1.62	0.03	0.05	0.00	0.02	0.06
1.63	0.02	0.07	0.01	0.02	0.04
1.65	0.04	0.07	0.01	0.02	0.05
1.67	0.03	0.03	0.01	0.02	0.05
1.69	0.04	0.01	0.01	0.01	0.04
1.70	0.04	0.01	0.01	0.02	0.03
1.72	0.03	0.04	0.01	0.02	0.04
1.74	0.04	0.05	0.01	0.02	0.05
1.75	0.03	0.04	0.00	0.02	0.05
1.77	0.03	0.03	0.00	0.01	0.04
1.79	0.04	0.02	0.01	0.01	0.04
1.80	0.03	0.02	0.01	0.01	0.03
1.82	0.02	0.02	0.00	0.01	0.03
