# Supplementary material for: Fallville: A Perspective on an Interactive Pedagogical Tool to Enhance Understanding and Implementation of Fall-Compliant Flooring
Source: Bioengineering (Basel). 2026 Jan 12;13(1):80. doi: 10.3390/bioengineering13010080 (PMC12838354; doi:10.3390/bioengineering13010080)
Supplement: Supplementary file 1 [file bioengineering-13-00080-s001.zip › Supplementary file Bioengineering S2.pdf]

	Hard	Soft
Tennis ball	Video S1	Video S2
Table tennis ball	Video S3	Video S4
Bouncy ball	Video S5	Video S6

QR codes for video recordings of ball drop experiment tracking
